# The role of rice husk in *Oreochromis niloticus* safety enhancement by bio-adsorbing copper oxide nanoparticles following its green synthesis: an endeavor to advance environmental sustainability

**DOI:** 10.1038/s41598-024-74113-0

**Published:** 2024-10-10

**Authors:** Aliaa Hamed, Shereen R. Badran

**Affiliations:** 1 Department of Biology, Basic Science Center, Misr University for Science and Technology (MUST), Giza, Egypt; 2 Department of Zoology, Faculty of Science, Cairo University, Giza, Egypt

**Keywords:** Sustainability. Green synthesis. Copper oxide nanoparticles. Bio-adsorbent. Rice husk. *O. Niloticus*, Ecology, Environmental sciences

## Abstract

Lowering nanoparticles (NPs) toxicity before discharge into aquatic environments and employing agricultural waste materials for environmental sustainability are necessary nowadays. Since this has never been done, this work examines how green CuO NPs treated with rice husk (RH) as a bio-adsorbent may be safer for Nile tilapia (*Oreochromis niloticus*) than chemically manufactured ones. So, five groups of fish were randomly placed in glass aquaria. One group was a control, and four groups received 50 mg/L green and chemically produced CuO NPs (GS and CS) with and without RH for 24, 48, and 96 h. RH was collected from all groups, and the results showed GS-CuO NPs had a greater adsorptive capacity than CS-CuO NPs after all time intervals. After analyzing fish indicators in all groups compared to the control, higher Cu bioaccumulation was exhibited in the liver and gills. The liver and gills showed elevated levels of glutathione peroxidase (GPx), catalase (CAT), and thiobarbituric acid reactive substances (TBARS), while the levels of glutathione reduced (GSH) were significantly lower. In addition, Cu exposure impaired liver and gill histology. Finally, our results indicated that using RH as an adsorbent for CuO NPs after their green synthesis instead of chemical synthesis before they enter the aquatic environment can enhance the overall health of fish and environmental sustainability.

## Introduction

## Materials and methods

### Fish adaptation period and rearing conditions

In well-aerated tanks, the mature male fish under study were transported to the ecology laboratory at Cairo University’s Faculty of Science from an unpolluted ranch in Kafr El-Sheikh. Following that, they were arranged at random in 40 L dechlorinated glass aquaria with constant aeration and dimensions of 40 × 70 × 26 cm for ten days of adaptation, and the water was changed daily at a rate of 30% to get rid of waste and extra food. The water’s parameters included a 25 ± 1 °C temperature, a pH of 7.2–7.4, ammonia levels of 0.22–0.30 mg/L, and a dissolved oxygen content of 6.6–7.9 mg/L. During this time, fish were given commercial pellet meals, which included crude (protein 32%, fiber 5%, fat 4%, ash 13%, and moisture 10%) daily at a rate of 3% of body weight. All fish feed until they reach apparent satiation, as NRC^32^ mentioned.

## Synthesis and characterization of CuO NPs

In our previous work^31^, GS-CuO NPs and CS-CuO NPs were produced, consistent with Nwanya et al.^33^ and Zhu et al.^34^. After that, both synthesized NPs were characterized, following Yugandhar et al.^35^, Joshi et al.^36^, Sharma et al.^37^, Akintelu et al.^38^, and Chandrasekaran et al. ^39^, as shown in Table 1.

**Table 1:** Characteristics summary of (GS and CS) CuO NPs.

Instrument	Reason for usage	Obtained results	
		GS-CuO NPs	CS-CuO NPs
UV-vis-spectrophotometer (UV-1800, Shimadzu, Japan).	To confirm the formation of the synthesized CuO NPs.	Revealed an absorption wavelength of 260 nm which confirmed its formation.	Revealed an absorption wavelength of 258 nm which confirmed its formation.
Field emission transmission electron microscopy (FETEM, JEM-2100 F, JEOL Inc., Japan).	To analyze the size and morphology.	Displayed that the synthesized CuO NPs were spherical, having a size < 50 nm.	
Scanning electron microscopy (FEI-SEM, Inspect S50, Netherlands) along with an energy dispersive X-ray spectrophotometer (EDX).	To provide shape, and compositional information on the presence of Cu and O_2_ elements.	They were relatively spherical and highly pure, as Cu and O were the main elements, and no irrelevant peaks were detected.	
Dynamic light scattering (DLS) (Nano-Zeta Sizer-HT, Malvern Instruments, UK).	To assess the CuO NPs average hydrodynamic size in water.	259.4 nm	218.3 nm
Zeta potential (Nano-Zeta Sizer-HT, Malvern Instruments, UK).	to assess the CuO NPs stability in water.	-10 mV	19.5 mV

## Preparation of RH

Before usage, the RH was divided into small pieces, cleaned with deionized distilled water to remove impurities, and dried for 24 h at 80 °C in the oven^23,40^. Then, the RH was ground and went through different sieves to give a particle size of 80 μm, which was chosen according to Naik et al.^29^.

## Experimental setup and exposure groups

**Fig. 1:**
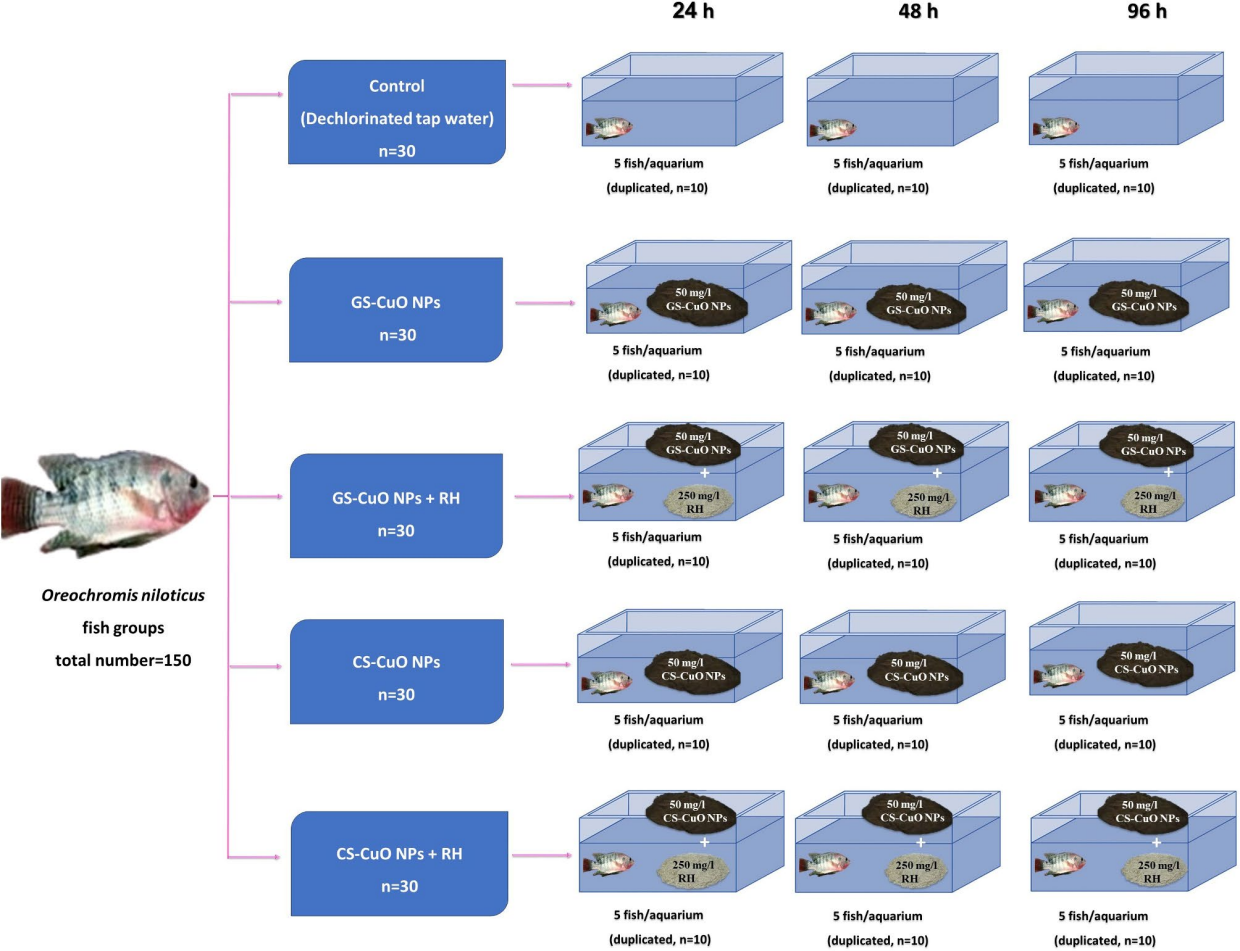
The present study’s experimental design.

The appropriate quantities of stock solutions were stirred at 300 rpm for 20 min to prevent aggregation, then added to the experimental aquaria to acquire concentrations of 50 mg/L of GS and CS CuO NPs for *O. niloticus* (50% LC_50_)^41^. As recommended by Abdel-Khalek et al.^23^, Khalil et al.^27^, and Naik et al.^29^, 250 mg/L of RH (5 times the NPs concentration) was added to fish aquaria and separated by nets with pores that allowed movement of synthesized CuO NPs without escaping into the water to prevent fish from eating them. Once the fish adaptation period ended, 150 healthy fish with a body length of 14 ± 0.76 cm and a weight of 39 ± 1.5 g were selected and randomly assigned into five main exposure groups. Each group contained 30 fish and was divided into three-time intervals of 24, 48, and 96 h with a stocking density of 5 fish per 40 L well-aerated glass aquarium (40 × 70 × 26 cm) in duplicated aquaria (as 10 fish for each time interval). As follows: first groups (control groups); second groups exposed to 50 mg/L GS-CuO NPs^41^; third groups exposed to 50 mg/L GS-CuO NPs + 250 mg/L RH^23,27,29^; fourth groups exposed to 50 mg/L CS-CuO NPs^41^; fifth groups exposed to 50 mg/L CS-CuO NPs + 250 mg/L RH for 24, 48, and 96 h, as shown in Fig. 1. During the experimental period, water was changed daily and re-dosed with synthesized CuO NPs to ensure water quality and exposure consistency. In addition, water conditions were monitored continually to match the acclimation period.

## RH collection and removal efficiency analysis

Following each experimental interval of 24, 48, and 96 h, RH (*n* = 4) was collected from the aquaria under study. RH was subjected to scanning electron microscopy (FEI-SEM, Inspect S50, Netherlands) and an energy dispersive X-ray-spectrophotometer (EDX) to assess Cu NPs on its surface. Additionally, the concentrations of Cu were evaluated on RH using an inductively coupled plasma instrument (ICP, AES, iCAP6000 series) made by Thermo Science. The removal efficiency of RH for GS-Cu and CS-Cu was calculated as follows: [%R = C_0_/C *100]^42^; where %R is the removal efficiency of RH, C_0_ is the initial Cu concentration in water, and C is the Cu concentration on RH after adsorption time.

### Fish and tissue sampling

Fish (*n* = 8) from each group were gathered randomly from experimental aquaria after 24, 48, and 96 h, anesthetized by immersion with 20 mg/L clove oil, and overdose (200 mg/L) was used to euthanize the fish^43^. After fish dissection, the liver and gills were removed and divided into portions allocated to Eppendorf microtubes. One portion of tissues was stored at -20 °C to determine the Cu levels. According to the kit instructions (Biodiagnostic, Dokki, Giza, Egypt), the other portion was washed with 0.9% cold NaCl and homogenized with phosphate buffer. Then, they were put in cooled centrifugation at 4000 rpm for 15 min. Afterward, the supernatants were accurately collected and preserved at -80 °C until the oxidative stress analyses were performed. For the histological procedures, a portion of liver and gill tissues were held in plastic tissue holders and cleaned with NaCl (0.7%), followed by an immersion into Bouin’s fixative.

## Detection of copper residues in fish liver and gills

By using the dry ashing method, the amount of Cu bioaccumulated inside the liver and gills (mg/kg) was measured using an inductively coupled plasma atomic emission spectroscopy instrument (iCAP, 6000 series, Thermo Scientific)^44^. First, the tissues were dried in an oven at 80 °C for 8 h; then, they were digested using 3 mL of concentrated hydrochloric acid (HCl). After that, the final product was diluted to a 25-mL volume using deionized water. Throughout the test, procedure blanks were used to ensure the correctness of the measurement and to adjust for background absorption. The National Institute of Standards and Technology (USA) standard reference material (Lake Superior Fish, 1946) was used to verify the analytical procedure. Recoveries of Cu were 95–100%. Additionally, as mentioned by Authman and Abbas^45^, Cu bioaccumulation factors (BAFs) were calculated as follows: BAFs are equal to the Cu concentration inside the studied tissues (mg/kg), divided by their concentration in water (mg/L).

## Determination of antioxidant biomarkers

The protocol for detecting all antioxidant indicators was followed (Biodiagnostic, Dokki, Giza, Egypt). The GPx activity measurement was consistent with the Paglia and Valentine^46^ method, in which their activity was indirectly determined by mixing 10µ of the sample with 100µ of hydrogen peroxide (H_2_O_2_) and a solution containing 100µ of (glutathione, glutathione reductase, and NADPH). In this reaction, glutathione reductase converts the oxidized glutathione back to its reduced form after GPx reduction. GP_X_ activity was linked to NADPH being oxidized to NADP^+^, resulting in a decrease in absorbance at 340 nm per min. The estimation of CAT activity followed the Aebi^47^ methodology. With this approach, 50µ of the sample was mixed with (100µ H_2_O_2_ and 500µ phosphate buffer) solution, which caused CAT to react with H_2_O_2_. After 1 min, the reaction was inhibited by adding a 200µ catalase inhibitor. Then, a chromophore was produced by the reaction between the H_2_O_2_ residues and 500 µ of (4-aminophenazone and 3,5-dichloro-2-hydroxybenzene sulfonic acid) after their addition with peroxidase to the reaction solution. The color intensity of the produced chromophore was measured at 510 nm absorbance, which is inversely proportional to the quantity of CAT in the sample. Every enzyme listed above is expressed as U/gr.protein. In the case of measuring lipid peroxidation in samples, the amounts of TBARS (nmol/gr.tissue) were an essential metric for assessment. According to Ohkawa et al.^48^, TBARS was produced when 1 mL of thiobarbituric acid and 200 µ of malondialdehyde in the sample were reacted for 30 min at 95 °C. By using spectrophotometry, the strength of the pink color produced was associated with the amounts of TBARS in the samples at 534 nm absorbance. Furthermore, Beutler et al.^49^ reported that the method for determining GSH (mmol/gr.protein) involved combining the 100µ sample with 500µ trichloroacetic acid, keeping it for 5 min at room temperature, then centrifuging it for 15 min at 3000 rpm. Additionally, 500µ of the supernatant was combined with 1 mL of buffer, and 500µ of 5,5′-dithiobis (2-nitrobenzoic acid) was added to cause a reduction. This reaction resulted in a yellowish color measured at 405 nm absorbance.

### Histological study

According to Suvarna et al.^50^, liver and gill tissues were dried, inserted in paraffin wax, and sectioned via a microtome after the fixation period. Hematoxylin and eosin were the last steps applied to the tissues. Finally, the histological alternations of each group’s tissue specimens were observed using a light microscope.

### Statistical analysis

All results were statistically examined using analyses of variance (ANOVA). Additionally, similarity measures within the various groups were determined using Duncan’s multiple ranges test, which was shown by a variety of upper and lower cases arranged in decreasing sequence. The mean ± SE (standard error) was used to display the data, and the mean difference was significant if the p-value was less than 0.05. Data analysis was examined using IBM’s SPSS 16.0 statistical program (Chicago, IL).

### Ethics statement

All methods were performed in accordance with the relevant guidelines and regulations. The present investigation was conducted in compliance with ARRIVE guidelines and approved by the Institutional Animal Care and Use Committee (IACUC), Faculty of Science, Cairo University, with approval no. (CUIF 1123).

## Results

### RH removal efficiency

**Fig. 2:**
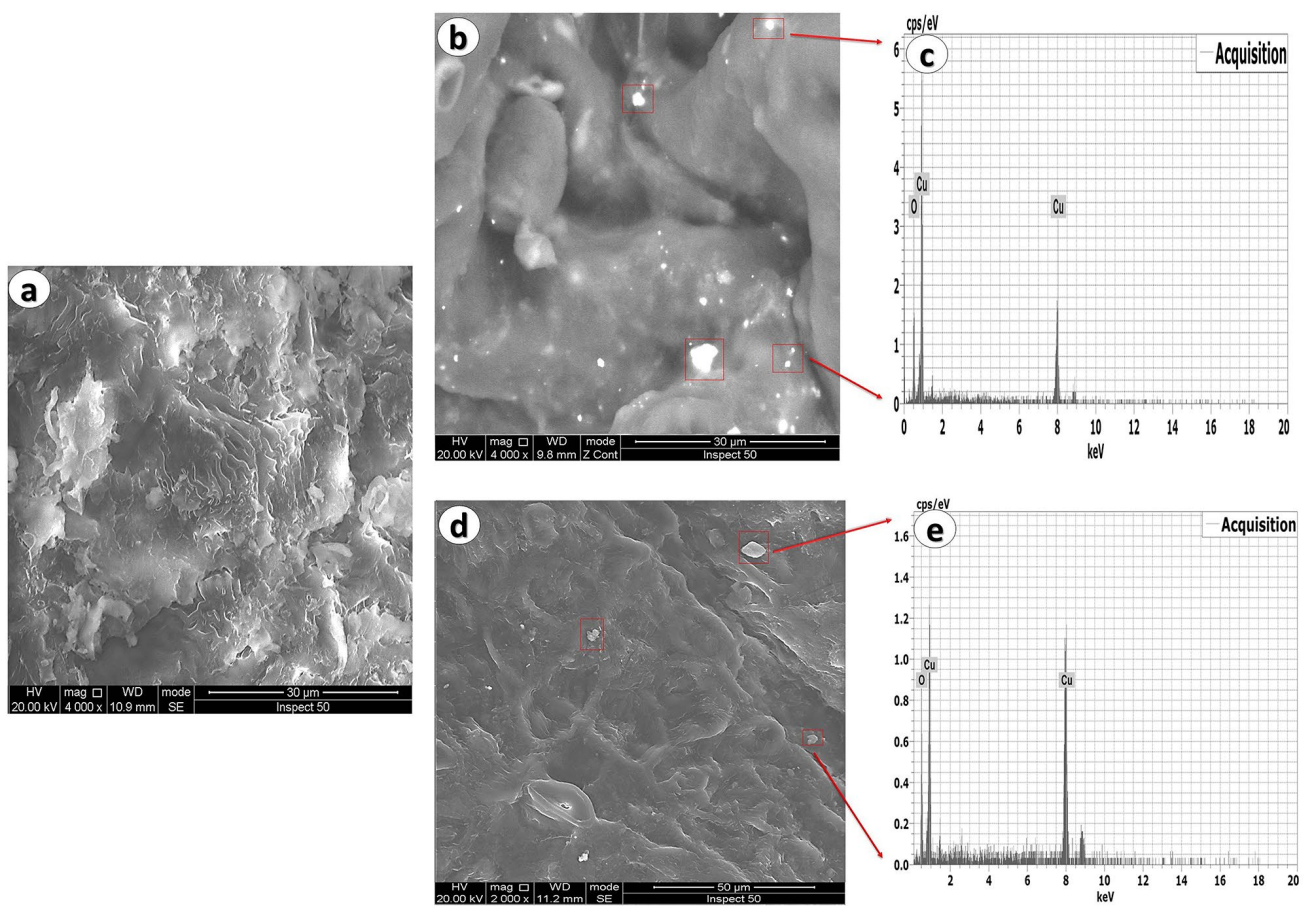
Representative RH surface analysis (**a**) SEM of RH before bio-adsorption; (**b**) SEM of RH after GS-Cu NPs bio-adsorption; (**c**) EDX spectra of RH after GS-Cu NPs bio-adsorption; (**d**) SEM of RH after CS-Cu NPs bio-adsorption; (**e**) EDX spectra of RH after CS-Cu NPs bio-adsorption.

**Fig. 3:**
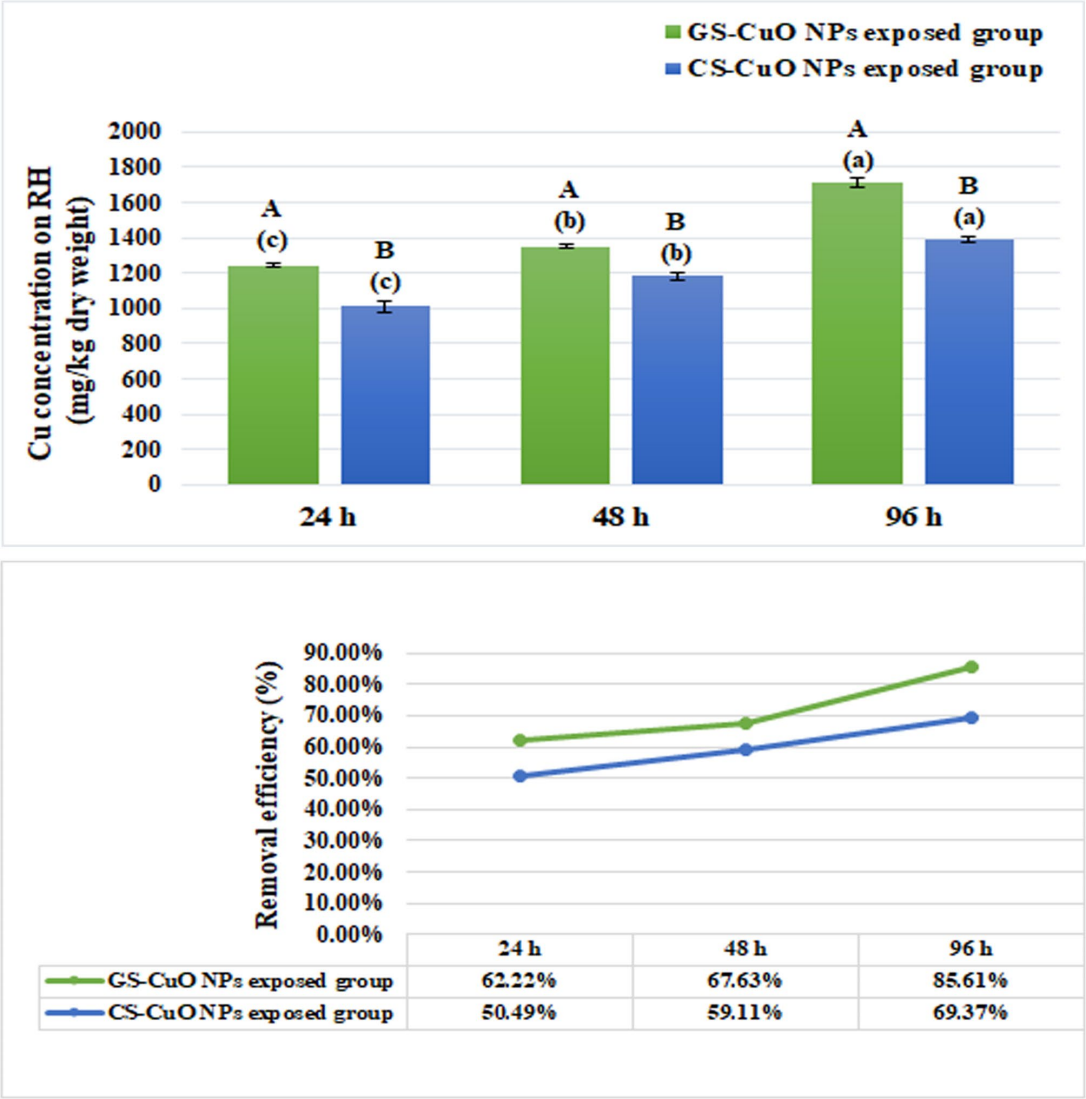
The concentration of Cu on RH and the removal efficiency of RH after 24, 48, and 96 h. All values are indicated as mean ± SE (*n* = 4). Letters A and B show statistically significant differences between the groups exposed to (GS and CS) CuO NPs after each time interval. Letters a, b, and c show statistically significant differences among the different times for each synthesized CuO NPs group. Values sharing common letters do not noticeably differ from each other.

Figure 2a SEM revealed a distinct and unaltered RH surface with no adsorption of Cu NPs. Following the exposure to GS and CS CuO NPs, the RH surfaces were observed to be coated with Cu NPs, which corroborated by the EDX spectra (Fig. 2b, c, d, and e). Figure 3 also represents the concentration of Cu on RH and the removal efficiency of RH after 24, 48, and 96 h. The concentration of Cu on RH and removal efficiency increased significantly (*p* < 0.05) with increasing time intervals, with the most significant elevation recorded after 96 h of GS-CuO NPs exposure.

### **Distribution of Cu in the studied tissues**

**Fig. 4:**
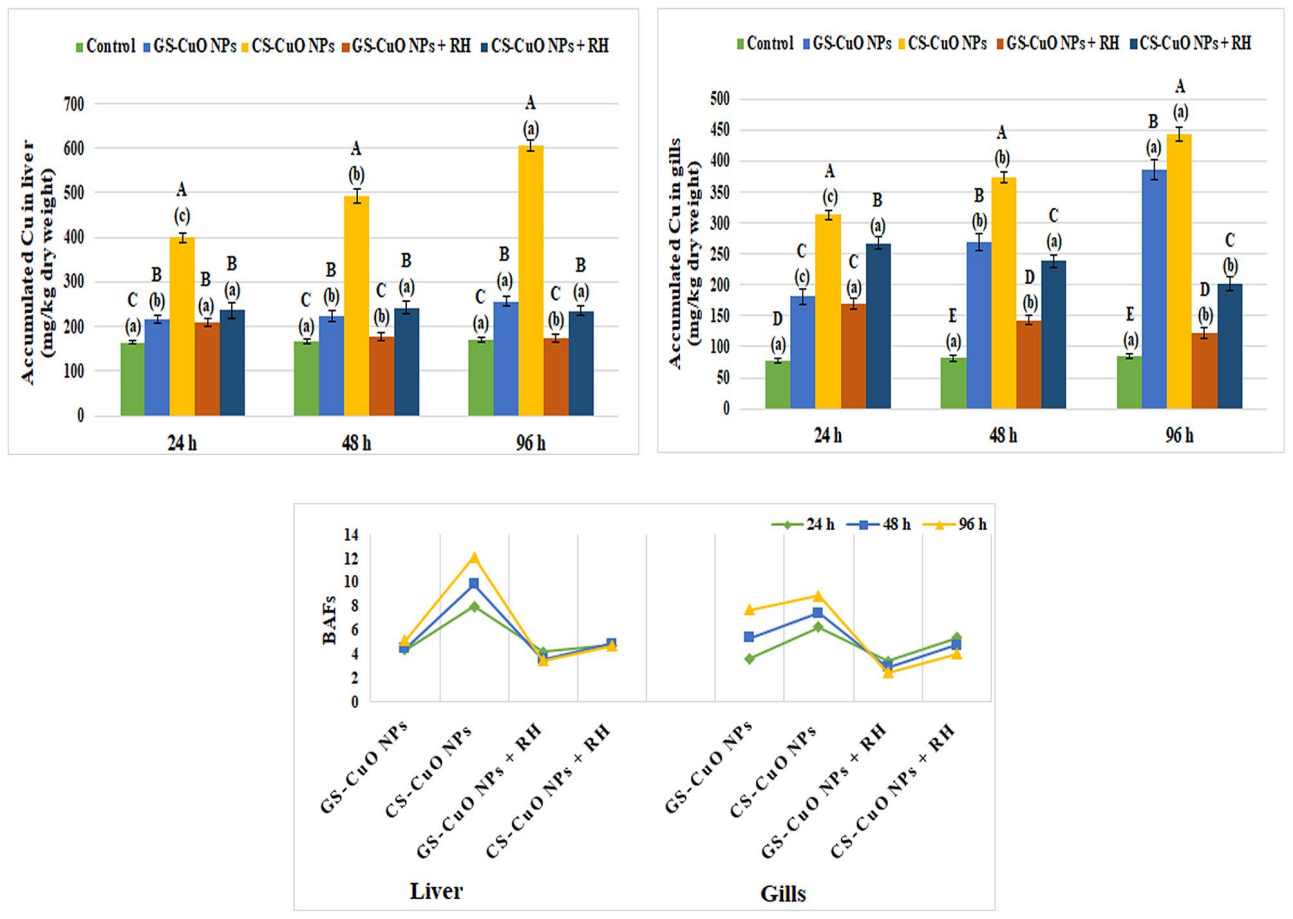
The bioaccumulation capacities of Cu and bioaccumulation factors (BAFs) in the liver and gills of *O. niloticus* exposed to 50 mg/L of GS-CuO NPs and CS-CuO NPs with and without RH water treatment after 24, 48, and 96 h. All values are indicated as mean ± SE (*n* = 8). Letters A, B, C, D, and E show statistically significant differences between the groups for each time, compared with the control group. Letters a, b, and c show statistically significant differences among different times for each group. Values sharing common letters do not noticeably differ from each other.

Figure 4 shows the ability of Cu to bioaccumulate and BAFs in the liver and gill tissues. A significant increase (*p* < 0.05) in Cu bioaccumulation was seen in the tissues of fish exposed to both CuO NPs with and without RH water treatment compared to the control groups. The concentration of Cu significantly rose (*p* < 0.05) proportionally with the duration of exposure in groups without RH treatment. Moreover, the Cu entrance percentages in the studied tissues decreased in the presence of RH and sharply decreased, particularly after 96 h of exposure to GS-CuO NPs.

### Antioxidant response of fish

**Fig. 5:**
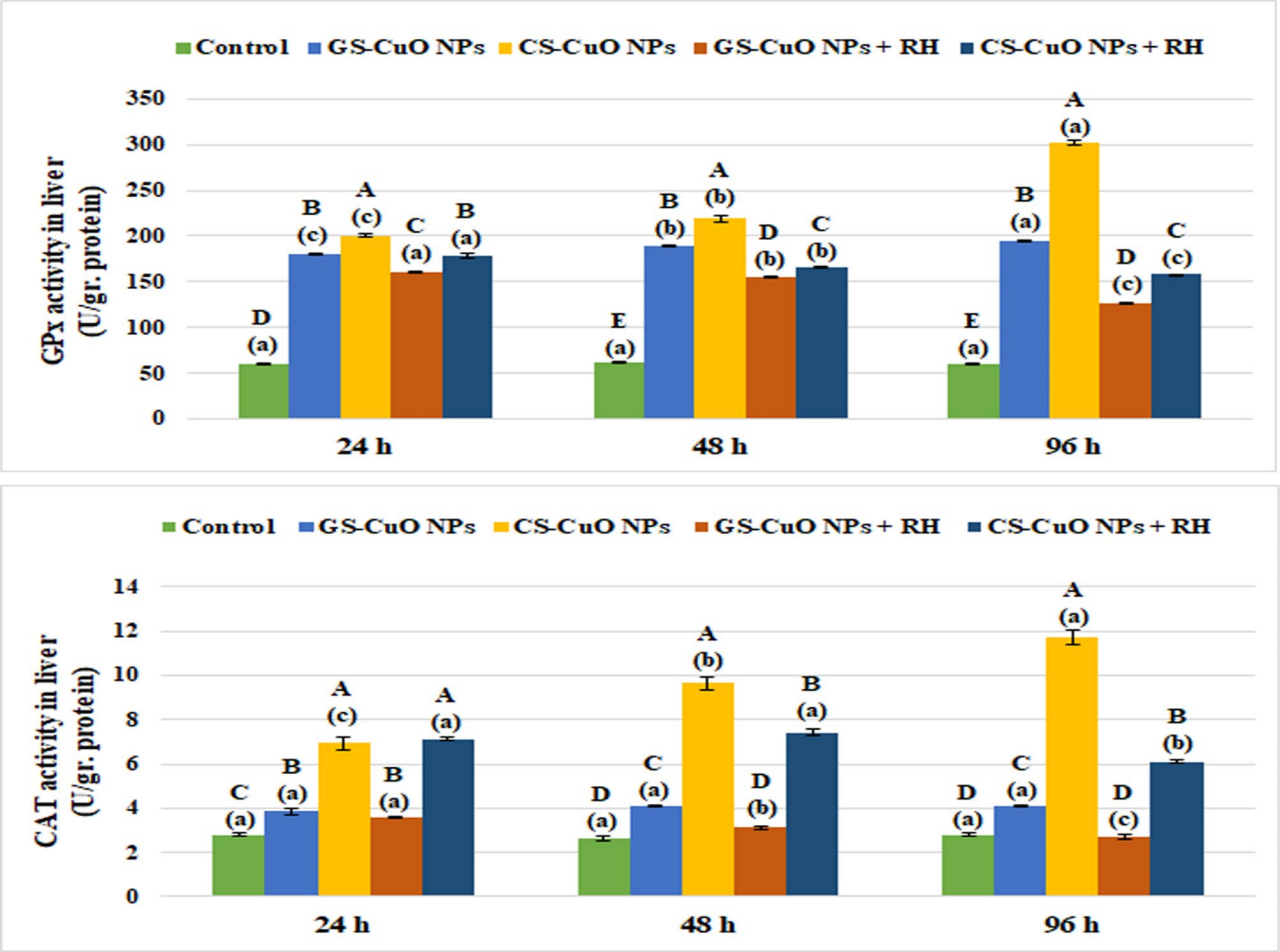
Changes in the liver GPx and CAT of *O. niloticus* groups exposed to 50 mg/L of GS-CuO NPs and CS-CuO NPs with and without RH water treatment after 24, 48, and 96 h. All values are indicated as mean ± SE (*n* = 8). Letters A, B, C, D, and E show statistically significant differences between the groups for each time, compared with the control group. Letters a, b, and c show statistically significant differences among different times for each group. Values sharing common letters do not noticeably differ from each other.

**Fig. 6:**
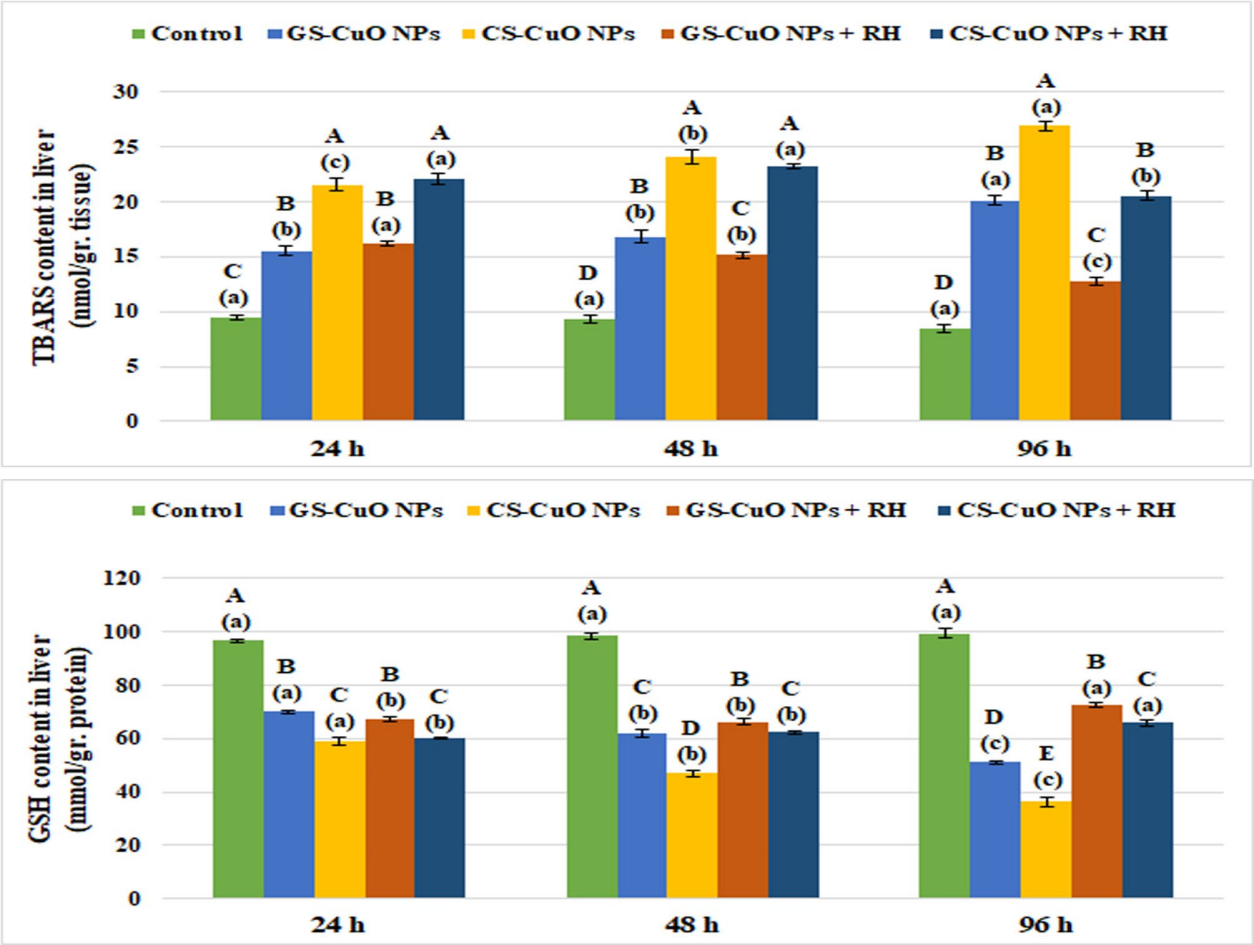
Changes in the liver TBARS and GSH of *O. niloticus* groups exposed to 50 mg/L of GS-CuO NPs and CS-CuO NPs with and without RH water treatment after 24, 48, and 96 h. All values are indicated as mean ± SE (*n* = 8). Letters A, B, C, D, and E show statistically significant differences between the groups for each time, compared with the control group. Letters a, b, and c show statistically significant differences among different times for each group. Values sharing common letters do not noticeably differ from each other.

**Fig. 7:**
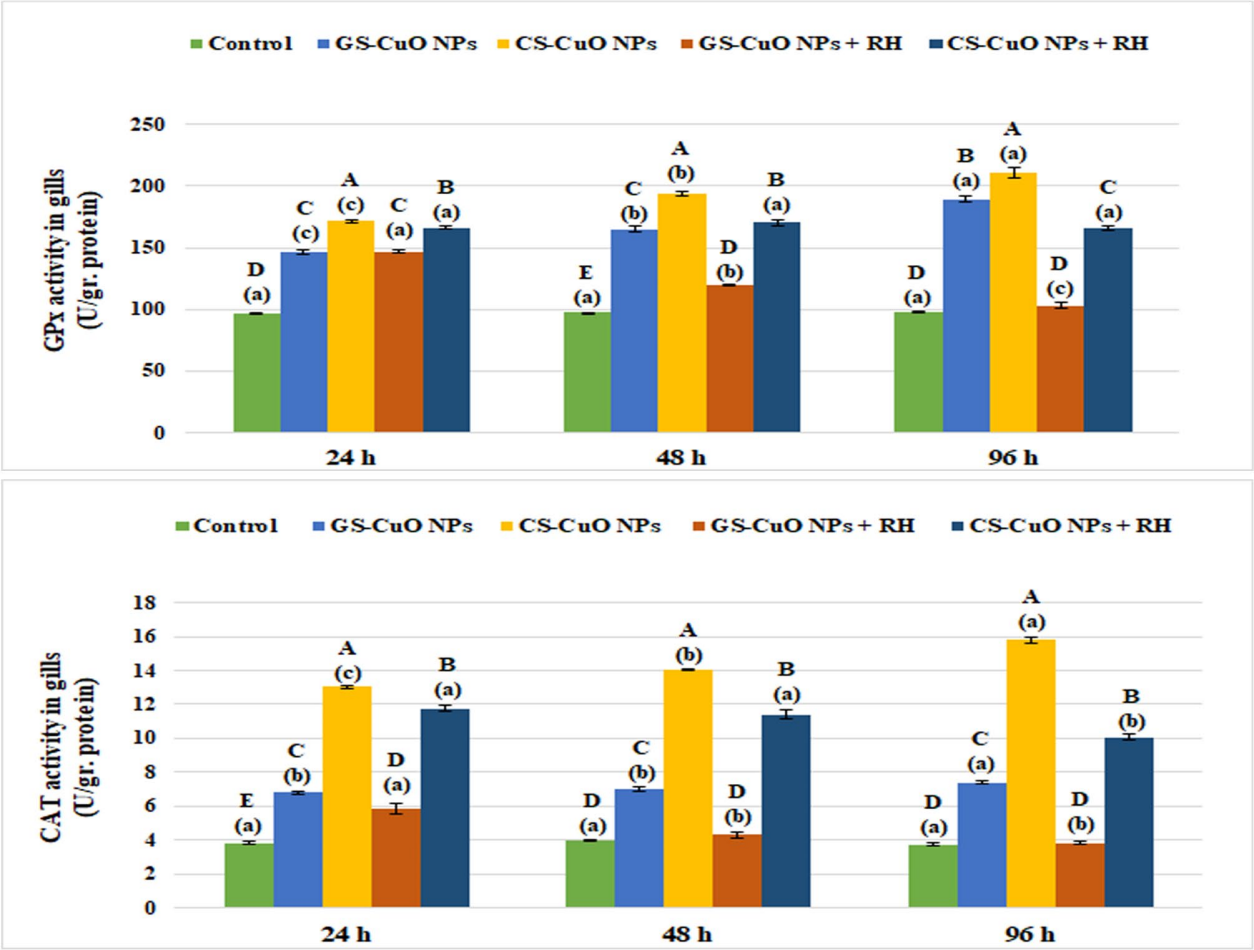
Changes in the gills GPx, and CAT of *O. niloticus* groups exposed to 50 mg/L of GS-CuO NPs and CS-CuO NPs with and without RH water treatment after 24, 48, and 96 h. All values are indicated as mean ± SE (*n* = 8). Letters A, B, C, D, and E show statistically significant differences between the groups for each time, compared with the control group. Letters a, b, and c show statistically significant differences among different times for each group. Values sharing common letters do not noticeably differ from each other.

**Fig. 8:**
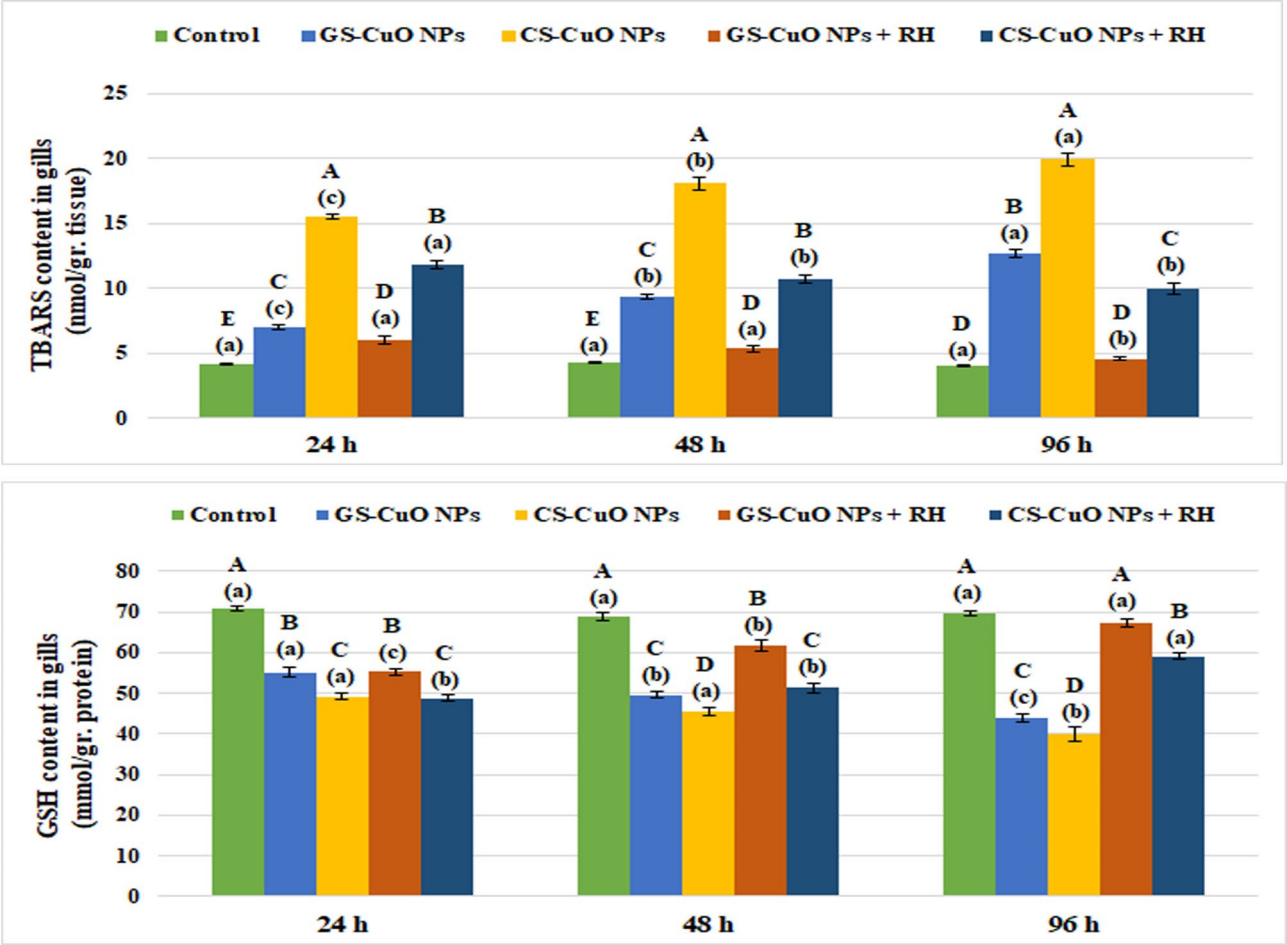
Changes in the gills TBARS and GSH of *O. niloticus* groups exposed to 50 mg/L of GS-CuO NPs and CS-CuO NPs with and without RH water treatment after 24, 48, and 96 h. All values are indicated as mean ± SE (*n* = 8). Letters A, B, C, D, and E show statistically significant differences between the groups for each time, compared with the control group. Letters a, b, and c show statistically significant differences among different times for each group. Values sharing common letters do not noticeably differ from each other.

Figure 5 shows that there was a notable increase (*p* < 0.05) in liver enzyme biomarkers (GPx and CAT) after 24, 48, and 96 h of exposure to GS and CS CuO NPs with and without RH water treatment. On the other hand, non-enzymatic antioxidant indicators (TBARS and GSH) showed a significant elevation (*p* < 0.05) in TBARS levels, and a significant decline (*p* < 0.05) in GSH levels (Fig. 6). Both enzymatic and non-enzymatic indicators in the gills displayed the same pattern as the liver (Figs. 7 and 8). After 96 h, all oxidative stress markers in the tissues exposed to CuO NPs without treatment showed the highest effects (*p* < 0.05). However, the presence of RH enhanced the oxidative biomarkers with increasing experimental time, particularly in the GS-CuO NPs groups.

### Histological findings

**Fig. 9:**
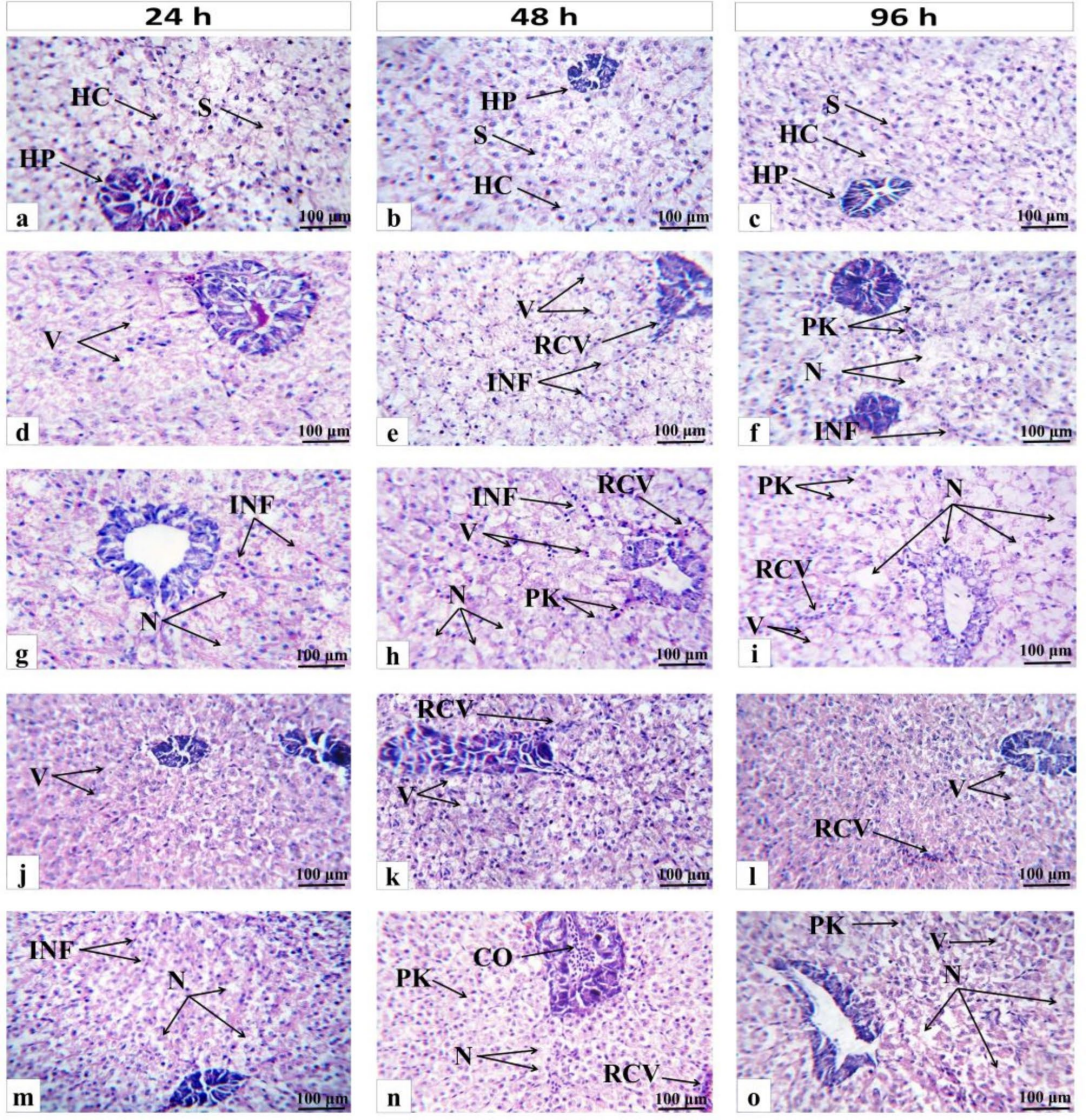
Representative histological liver alterations of *O. niloticus* after 24, 48, and 96 h, respectively. (**a**, **b**, **c**) the control groups; (**d**, **e**, **f**) 50 mg/L GS-CuO NPs exposed groups; (**g**, **h**, **i**) 50 mg/L CS-CuO NPs exposed groups; (**j**, **k**, **l**) 50 mg/L GS-CuO NPs + RH exposed groups; (**m**, **n**, **o**) 50 mg/L CS-CuO NPs + RH exposed groups. **HC**, hepatic cells; **HP**, hepatopancreas; **S**, sinusoids; **V**, cytoplasmic vacuolation; **RCV**, rupture of central vein; **INF**, infiltration of blood cells; **PK**, pyknotic nuclei; **N**, necrosis; **CO**, congestion in blood vessels.

**Fig. 10:**
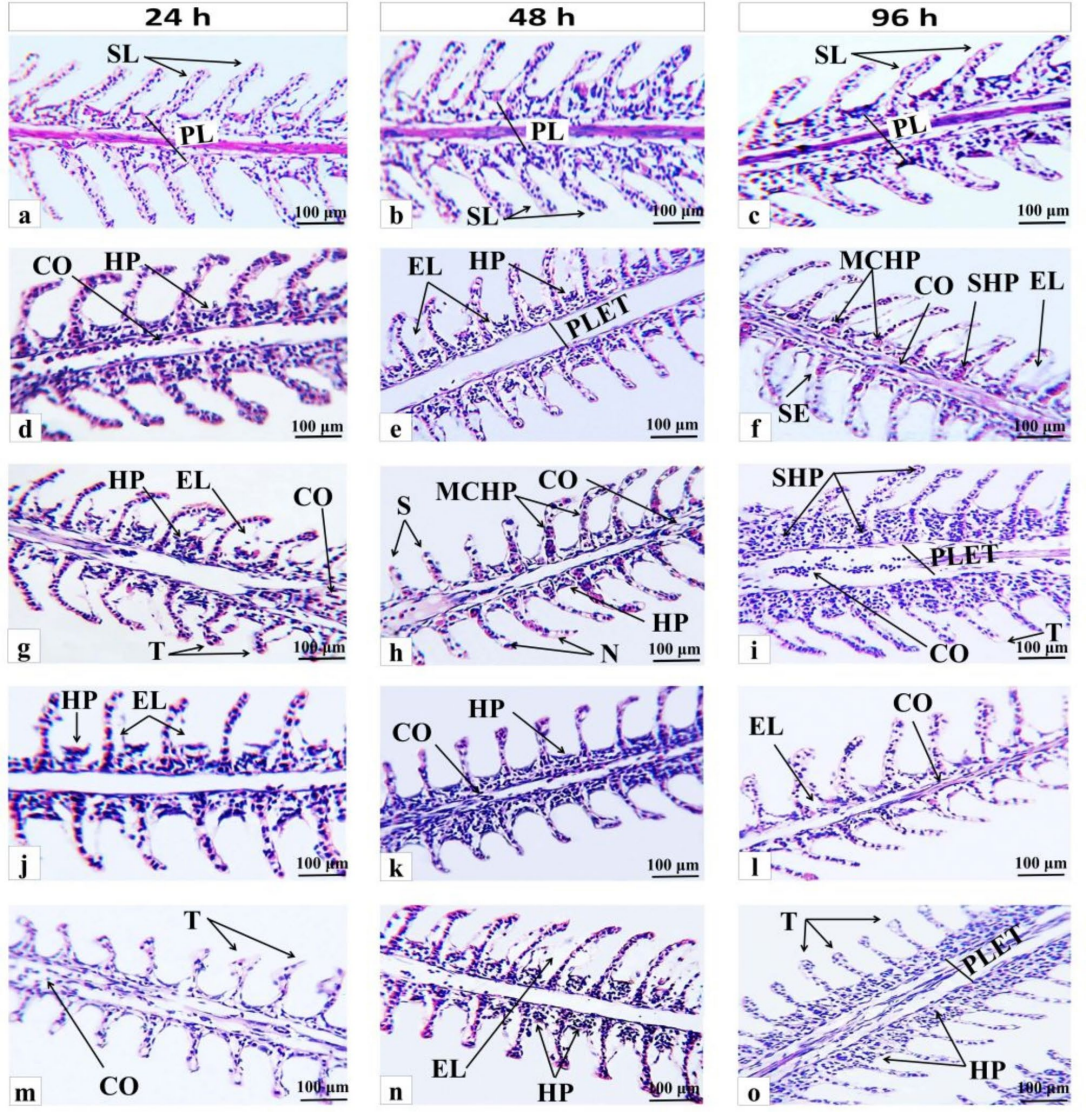
Representative histological gill alterations of *O. niloticus* after 24, 48, and 96 h. (**a**, **b**, **c**) the control groups; (**d**, **e**, **f**) 50 mg/L GS-CuO NPs exposed groups; (**g**, **h**, **i**) 50 mg/L CS-CuO NPs exposed groups; (**j**, **k**, **l**) 50 mg/L GS-CuO NPs + RH exposed groups; (**m**, **n**, **o**) 50 mg/L CS-CuO NPs + RH exposed groups. **PL**, primary lamellae; **SL**, secondary lamellae; **CO**, congestion in the lamellar blood vessels; **HP**, hyperplasia; **EL**, epithelial lifting; **PLET**, primary lamellar epithelial thickening; **MCHP**, mucosal cell hyperplasia; **SHP**, severe hyperplasia; **T**, telangiectases at the tip of secondary lamellae; **S**, shortening to secondary lamellae; **N**, necrosis.

The damaging impacts of both (GS and CS) CuO NPs on the histological architecture of the liver and gill of *O. niloticus* after 24, 48, and 96 h are displayed in Figs. 9 and 10, respectively. The control fish’s hepatic tissues (Fig. 9a, b, and c) displayed normal architecture with densely packed polygonal hepatocytes sinusoids scattered randomly throughout the hepatocytes, large central or sub-central sphere-shaped nuclei, and homogenous cytoplasm. The liver architecture of fish exposed to synthesized CuO NPs with and without RH for the periods studied (Fig. 9d to 8o) changed significantly. It was shown by changes in the liver’s tissues, such as vacuolization in the cytoplasm, congestion in blood vessels, rupture of the central vein, pyknotic nuclei, infiltration of blood cells, and necrosis. The gills of the control group (Fig. 10a, b, and c) also had well-organized primary lamellae and secondary lamellae that were well-spaced out and had flat epithelial cells at their bases. The histological changes in the gills were observed after 24, 48, and 96 h of (GS and CS) CuO NPs exposure without any treatment. Some of these changes were the thickening of the primary lamellar epithelium, lifting of the secondary lamellae epithelium, shortening of the secondary lamellae, telangiectases, an increase in the number of blood vessels in the lamellae, severe hyperplasia and necrosis of the primary lamellae (Fig. 10d, e, f, g, and h). Furthermore, fish gill sections exposed to both synthetic CuO NPs treated with RH at different experimental times (Fig. 10j, k, l, m, n, and o) demonstrated secondary lamellae epithelial lifting, primary lamellar epithelial thickening, lamellar blood vessel congestion, hyperplasia, and secondary lamellae telangiectases. In the present study, the severity of the alterations after exposure to CuO NPs without RH treatment increased with time. After RH water treatment, the histological alterations were less noticeable in all studied groups, especially after 96 h exposure to GS-CuO NPs.

## Discussion

The process of green synthesis has garnered considerable interest in material science due to its sustainable, reliable, and environmentally friendly nature. Several studies have demonstrated that NPs generated using green methods exhibit lower toxicity than those synthesized using chemical methods^30,51,52,53^. However, according to Ibrahim et al.^54^, this does not imply that green NPs are entirely safe for aquatic creatures. Thus, developing techniques to guarantee their safety in aquatic environments through their treatment before discharge with eco-friendly products can promote environmental sustainability.

Our study indicated that RH had a higher ability to remove CuO NPs from water. It might be due to its high cellulose, hemicellulose, lignin, and silica content, which rapidly binds metals, as well as its granular form and chemical stability, as stated by Abdel-Khalek et al.^23^. According to the current study, after 96 h of exposure, there was a considerable increase in the removal efficiency of Cu toward RH. It was consistent with the findings of Naik et al.^29^ and Zafar et al.^55^, who stated that the percentage removal of Cu was enhanced with time. It was caused by the maximum number of empty sites on RH and the interaction between adsorption sites and Cu over time. Additionally, the RH adsorption capacity for GS-CuO NPs was higher than CS-CuO NPs after all time intervals. It could be because of the different surface charges of the produced NPs^31^, which makes it easier for GS than CS CuO NPs to adsorb on the RH surface and aggregate around them.

## Conclusion

This study was the first to investigate the effect of using RH as a bio-adsorbent for CuO NPs after their green synthesis compared to those made chemically on *O. niloticus* at different periods. The current study revealed that GS-CuO NPs were less harmful to *O. niloticus* than CS-CuO NPs. Additionally, the toxicity of GS-CuO NPs significantly decreased after adding RH, indicating the most effective removal of GS-CuO NPs. It meant there was a good chance it could improve the fish’s health. To lower the harmful effects of NPs on aquatic environments, we suggest encouraging the use of RH as a bio-adsorbent for NPs following their green synthesis before they enter the water. Further research is required to enhance this approach, and alternative treatment methods can ensure their safety in aquatic ecosystems.

## Data Availability

The datasets used and/or analyzed during the current study are available from the corresponding author on reasonable request.
